# Vertical GaN Power MOSFET with Integrated Fin-Shaped Diode for Reverse Conduction

**DOI:** 10.1186/s11671-022-03717-0

**Published:** 2022-08-24

**Authors:** Tao Sun, Xiaorong Luo, Jie Wei, Kemeng Yang, Siyu Deng, Zhijia Zhao, Yanjiang Jia, Bo Zhang

**Affiliations:** State Key Laboratory of Electronic Thin Films and Integrated Devices, University of Electronic Science and Technology of China, Chengdu, 610054 China

**Keywords:** GaN, Vertical power MOSFET, Reverse conduction, Turn-on voltage, Gate charge, Reverse recovery, Fin

## Abstract

A vertical GaN power MOSFET featuring an integrated fin-shaped non-junction diode (FDMOS) is proposed to improve reverse conduction and switching characteristics. Its static and dynamic characteristics are studied and analyzed by Sentaurus TCAD simulation. Compared with the conventional MOSFET (Con. MOS) with a body diode as a freewheeling diode (FWD), the FDMOS uses the integrated fin-shaped diode to reverse conduction, and thus, a low reverse turn-on voltage *V*_ON_ of 0.66 V is achieved, with a decreasing of 77.9%. Moreover, the *Q*_rr_ of the FDMOS is reduced to 1.36 μC from 1.64 μC of the Con. MOS, without the minority carrier injection. The gate charge (*Q*_GD_) of the FDMOS is significantly reduced because the fin structure reduces the gate area and transforms some part of *C*_GD_ to *C*_GS_, and thus, a low switching loss is realized. The *Q*_GD_, the turn-on loss (*E*_on_) and the turn-off loss (*E*_off_) of the FDMOS are decreased by 56.8%, 33.8% and 53.8%, respectively, compared with those of the Con. MOS. In addition, the FDMOS is beneficial to reduce the parasitic inductance and the total chip area compared with the conventional method of using an externally connected Schottky diode as an FWD.

## Introduction

GaN-based devices are excellent candidates for power devices due to high critical electric field, high electron mobility and high-temperature operation [1–5]. Many researches focus on lateral high electron mobility transistors (HEMTs) because of its high-density and high-mobility two-dimensional electron gas (2DEG) [6–8], which allows high-voltage devices with low on-resistance and high switching speed. However, owing to the high-density interface states and high surface electric field (E-field) peak, the lateral HEMTs normally suffer from severe stability and reliability issues [9]. The vertical transistors can make full use of the potential of GaN material for high breakdown voltage [10–12].

For practical use of the vertical transistors in topologies of power converters (e.g., buck/boost converters), reverse conduction with low loss is also demanded to release the surplus energy induced by the inductive load [13]. The typical turn-on voltage (*V*_ON_) of the body diode in Si MOSFET is only 0.7 V, while the typical *V*_ON_ of the GaN PiN diode is close to 3 V [14]. Moreover, the P-i-N diode is bipolar device, and its rise time (*t*_r_) and fall time (*t*_f_) are very large, owing to the large reverse recovery charge (*Q*_rr_) during on/off transition [15, 16]. Using an external anti-paralleled Schottky barrier diode (SBD) as a freewheeling diode (FWD) for reverse conduction is easy to reduce *V*_ON_. However, the large parasitic inductance is introduced, resulting in the extra power loss and system instability. One solution is the monolithically integration of the SBD. It decreases the number of parasitic components and simplifies packaging [17], but the leakage current in the off-state is a drawback nevertheless.

In this paper, a novel vertical GaN MOSFET with integrated fin-shaped diode (FDMOS) is proposed to improve the reverse conduction characteristic. Compared with the conventional MOSFET (Con. MOS) with a body diode as a FWD, the *V*_ON_ and *Q*_rr_ of the FDMOS are significantly reduced. In addition, compared with the conventional method of using an externally connected Schottky diode as an FWD, the integrated fin-shaped diode doesn’t occupy the extra chip area and introduce parasitic inductance.

## Structure and Mechanism

**Fig. 1:**
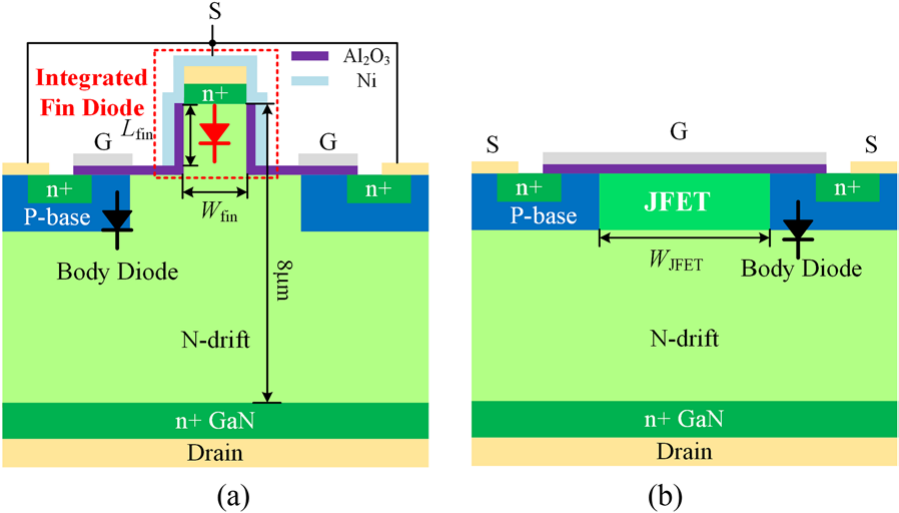
Schematic cross section of the **a** MOSFET with integrated fin diode (FDMOS). Two gates (G) are also shorted. **b** Conventional MOSFET (Con. MOS)

The schematic cross-sectional view of the FDMOS is proposed in Fig. 1a. Compared with the Con. MOS in Fig. 1b, the middle of the planar gate is replaced by a fin-shaped non-junction diode for reverse conduction. In the reverse conduction state, the fin-shaped diode turns on prior to the body diode and thus the reverse turn-on voltage (*V*_ON_) is greatly reduced. Moreover, without minority carrier injection, reverse recovery charge (*Q*_rr_) is reduced. Benefiting from the separated gate, the FDMOS exhibits a low *Q*_GD_. Simulations are carried out by Sentaurus TCAD. The thickness of the N-drift and the n+ GaN is 7 μm and 0.3 μm, respectively. The thickness of the Al_2_O_3_ is 20 nm. The metal work function is 5.15 eV. The doping concentrations of the N-drift (*N*_d_), JFET region and the n+ GaN are 1 × 10^16^ cm^−3^, 5 × 10^16^ cm^−3^ and 1 × 10^21^ cm^−3^. The *W*_JFET_ and *N*_JFET_ for the Con. MOS are 1 μm and 5 × 10^16^ cm^−3^, respectively. The simulation model used in this paper is similar to the REF [18], which was calibrated by experiment. The physical models include energy bandgap, incomplete ionization, electron and hole mobility, polarization, impact ionization, and radiative and non-radiative recombination. The electron mobility of the fin’s sidewall is assumed to be 13 cm^2^/V·s in TCAD.

**Fig. 2:**
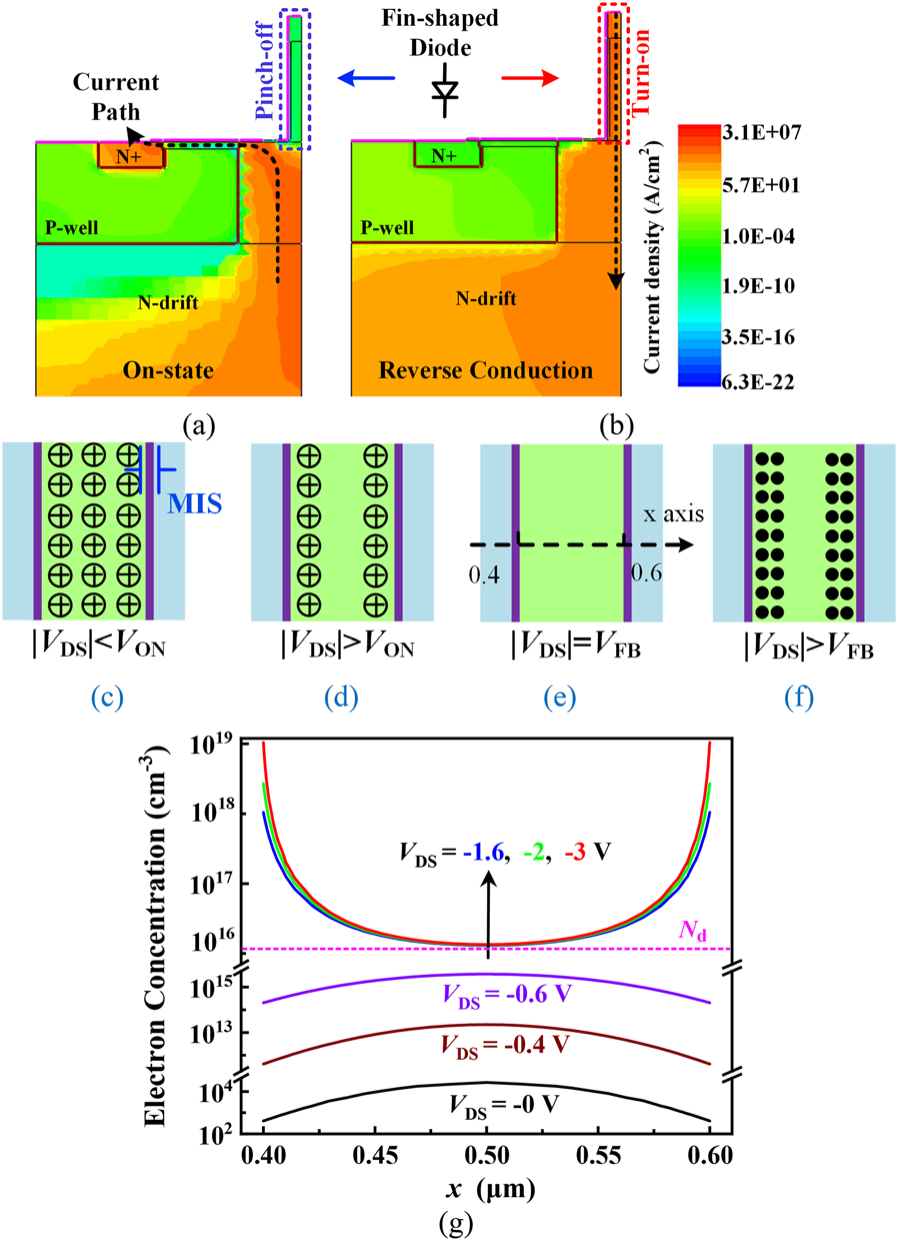
Simulated results of the FDMOS **a** on-state current contour and **b** reverse conduction current contour. **c**–**f** Operation mechanism analysis of the fin-shaped diode. **g** Electron concentration distribution along the cutline at different reverse voltages

The fin-shaped diode is pinched off at *V*_DS_ = 0 V because the fin is fully depleted by the work function difference of the MIS structure in the fin sides, as shown in Fig. 2a, c. Therefore, the fin-shaped diode does not affect the forward conduction characteristic. At reverse conduction state, the fin-shaped diode acts as an FWD. With an increasing reverse biased voltage, the depletion area shrinks, and the electron accumulation layer forms along the fin side wall, which provides a reverse current path, as shown in Fig. 2b, f. Figure 2g shows the electron concentration (*N*_e_) along a lateral cutline in the fin channel under different bias voltage *V*_DS_. The cutline is shown in Fig. 2e, which is in the middle of the fin. The change of *N*_e_ is consistent with the trend in Fig. 2c–f. The *N*_e_ > *N*_d_ near the sidewall verifies the formation of electron accumulation layer.

## Results and Discussion

**Fig. 3:**
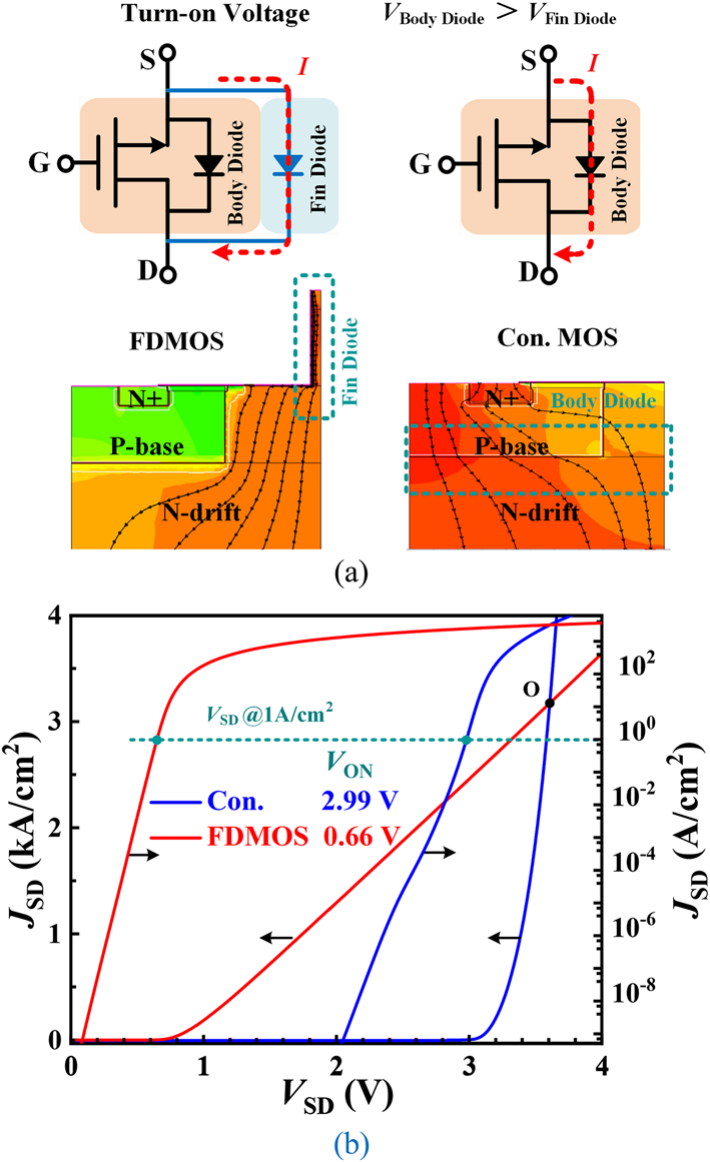
**a** Equivalent circuit and current density distribution (@*V*_DS_ =  − 4 V) of the reverse conduction. **b** Reverse conduction characteristics (log-scale and linear-scale) of the FDMOS and the Con. MOS

Figure 3 shows the equivalent circuit of the reverse conduction and reverse conduction characteristic. Figure 3a shows the current path and current density distribution at reverse conduction state. The reverse current in the FDMOS flows through the fin-shaped diode, while the current conduct by the body diode in Con. MOS. According to Fig. 3b, the fin-shaped diode as an FWD exhibits a much lower *V*_ON_ of 0.66 V (@1 A/cm^2^) in the FDMOS than 2.99 V of the Con. MOS. However, the current capacity (above point O) of the Con. MOS is higher than that of the FDMOS since the Con. MOS works in a bipolar conduction mode, which introduces greater reverse recovery loss nevertheless. The body diode doesn’t conduct because the voltage drop on the body diode for the FDMOS is lower than its turn-on voltage at the reverse conduction state.

**Fig. 4:**
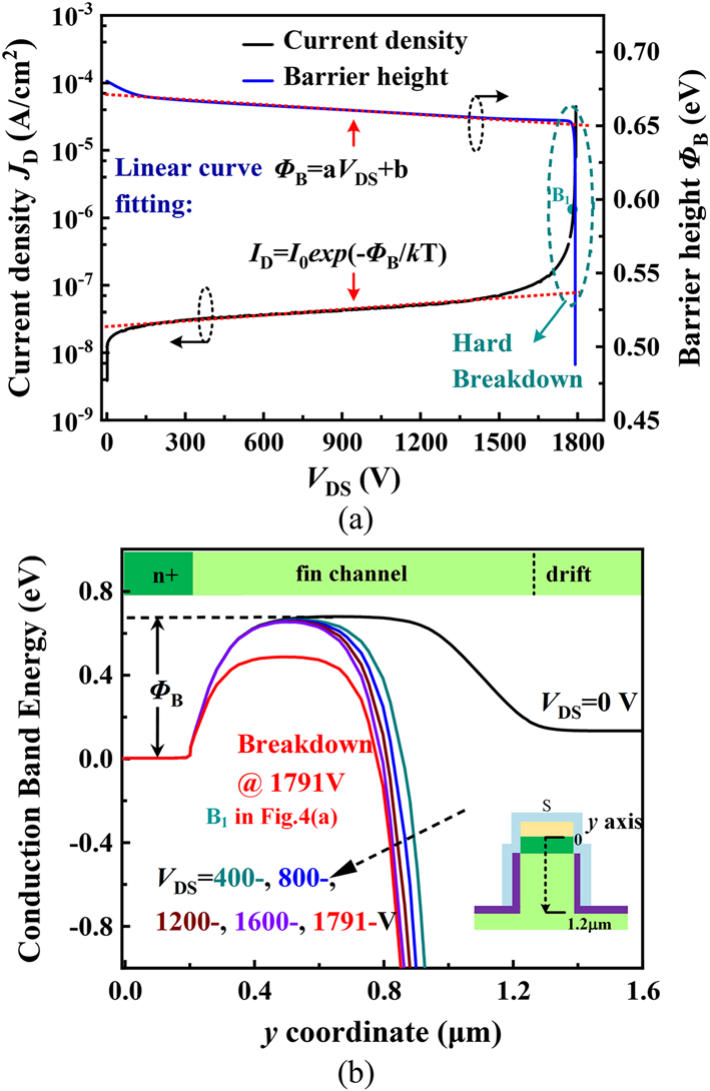
**a** Breakdown mechanism analysis of the fin-shaped diode. Linear curve fitting of the current density (*I*_D_) and barrier height (*Φ*_B_). The a and b are the coefficients. **b** Simulated conduction band energy extracted from the cutline at different *V*_D_ values

Figure 4 shows the analysis of breakdown characteristics. BV is defined as the *V*_DS_ @ 10^−6^A/cm^2^. As shown in Fig. 4a, the FDMOS achieves a hard avalanche breakdown voltage of 1791 V. The fin-shaped diode has a very low reverse leakage current of ~ 10^−7^ A/cm^2^ at *V*_DS_ =  − 1600 V, and the switching current ratio (*I*_on_/*I*_off_) is over 10^10^. The leakage current and the barrier height (*Φ*_B_) satisfy the I ∝ exp(− *Φ*_B_/*kt*) relation. Figure 4b shows the extracted conduction band energy along the middle of fin channel at different *V*_DS_ values. The width of barrier decreases with the increasing *V*_DS_, and the barrier height decreases almost linearly. The holes generated by the avalanche breakdown enter the fin channel region leads to the increase in the fin potential, and thus, the barrier height decreases rapidly. Therefore, the leakage current increases rapidly and the breakdown occurs.

**Fig. 5:**
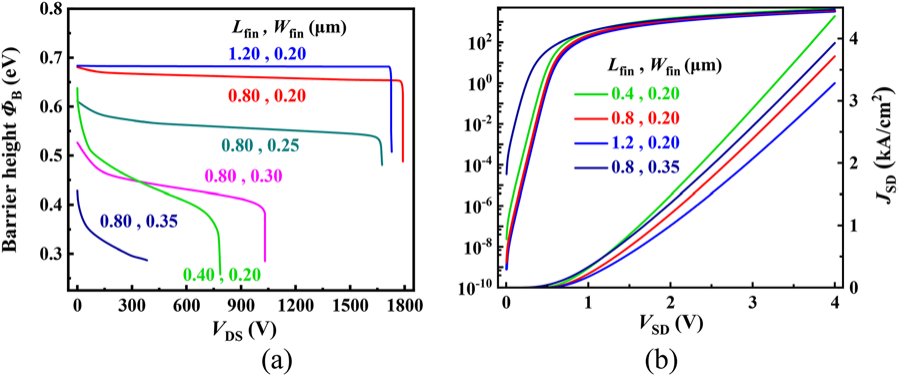
Influence of the *L*_fin_ and *W*_fin_ on **a** *Φ*_B_ and **b** *J*_SD_ for the FDMOS

Figure 5 shows the impacts of *L*_fin_ and *W*_fin_ on *Φ*_B_ and reverse conduction characteristics for the FDMOS. As shown in Fig. 5a, *Φ*_B_ decreases with the increase in *W*_fin_ and increases with the increase in *L*_fin_ because of the increasing overlap of the depletion region. A high *Φ*_B_ is beneficial to achieving a high breakdown voltage, but it leads to the high *V*_ON_. In addition, the fin channel mobility is low, and thus, the resistance increases with the increase in *L*_fin_, as shown in Fig. 5b. Considering the trade-off between breakdown characteristics and on-state performance, the optimized *L*_fin_ and *W*_fin_ is 0.8 μm and 0.2 μm, respectively.

**Fig. 6:**
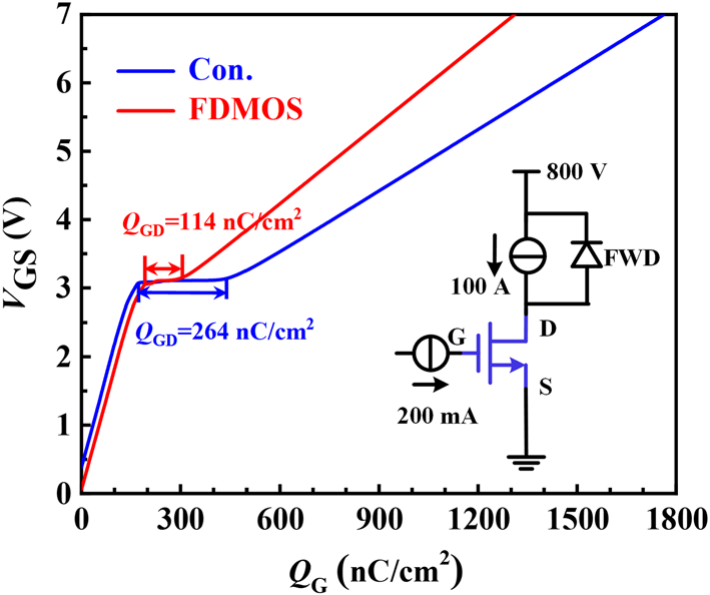
Simulated gate charges. The inset figure shows the test circuit

Figure 6 shows the gate charge characteristics of the two devices. The *Q*_GD_ of the FDMOS is 114 nC/cm^2^, which is far less than 264 nC/cm^2^ of the Con. MOS. One reason is that the overlap area between the gate and drain is reduced. The other is that the source metal surrounding the fin can effectively shield the gate-drain overlap, and thus, part of the capacitance between gate and drain (*C*_GD_) transforms to the capacitance between gate and source (*C*_GS_) for the FDMOS.

**Fig. 7:**
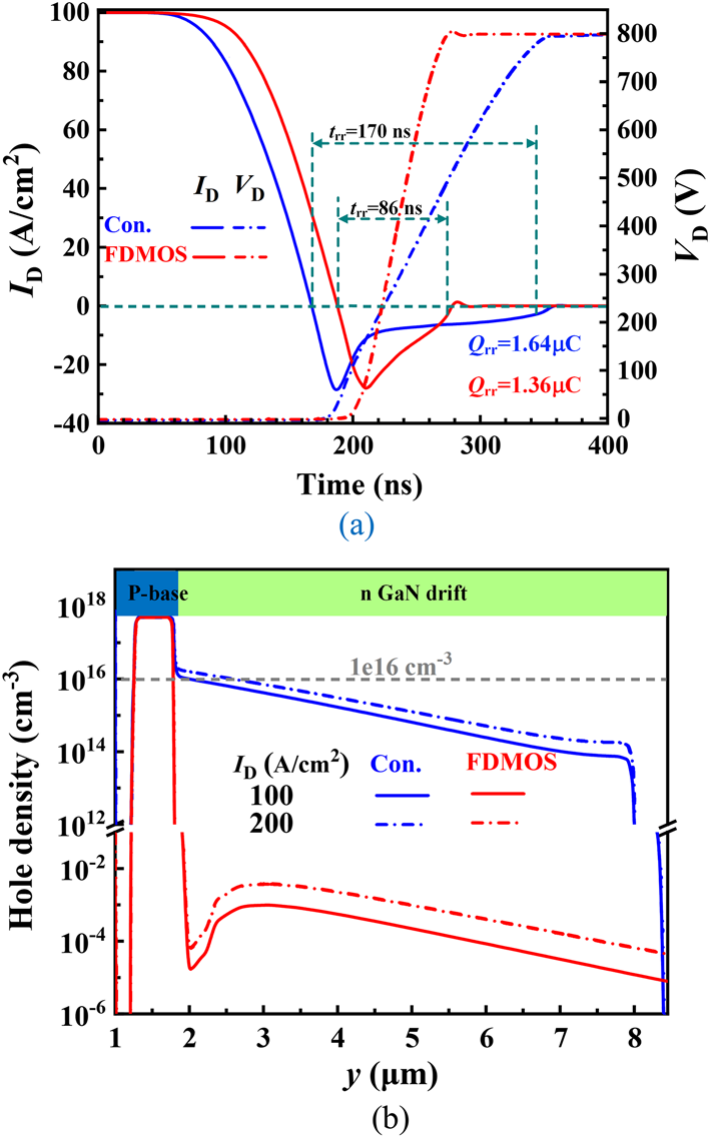
Comparison of **a** reverse recovery characteristic and **b** hole distribution during reverse recovery

Figure 7a shows that the FDMOS achieves better reverse recovery characteristics and lower reverse recovery loss. Figure 7b compares the hole distribution of the FDMOS and the Con. MOS during reverse recovery. The FDMOS device is in unipolar mode, and thus, the hole concentration in the drift region is very low, which is far less than n-drift concentration (1 × 10^16^ cm^−3^). However, the drift region of the Con. MOS has a high hole concentration due to the minority injection. Compared with the Con. MOS, the FDMOS reduces the *Q*_rr_ from 1.64 to 1.36 μC, and reduces *t*_rr_ from 170 to 86 ns as shown in Fig. 7a.

**Fig. 8:**
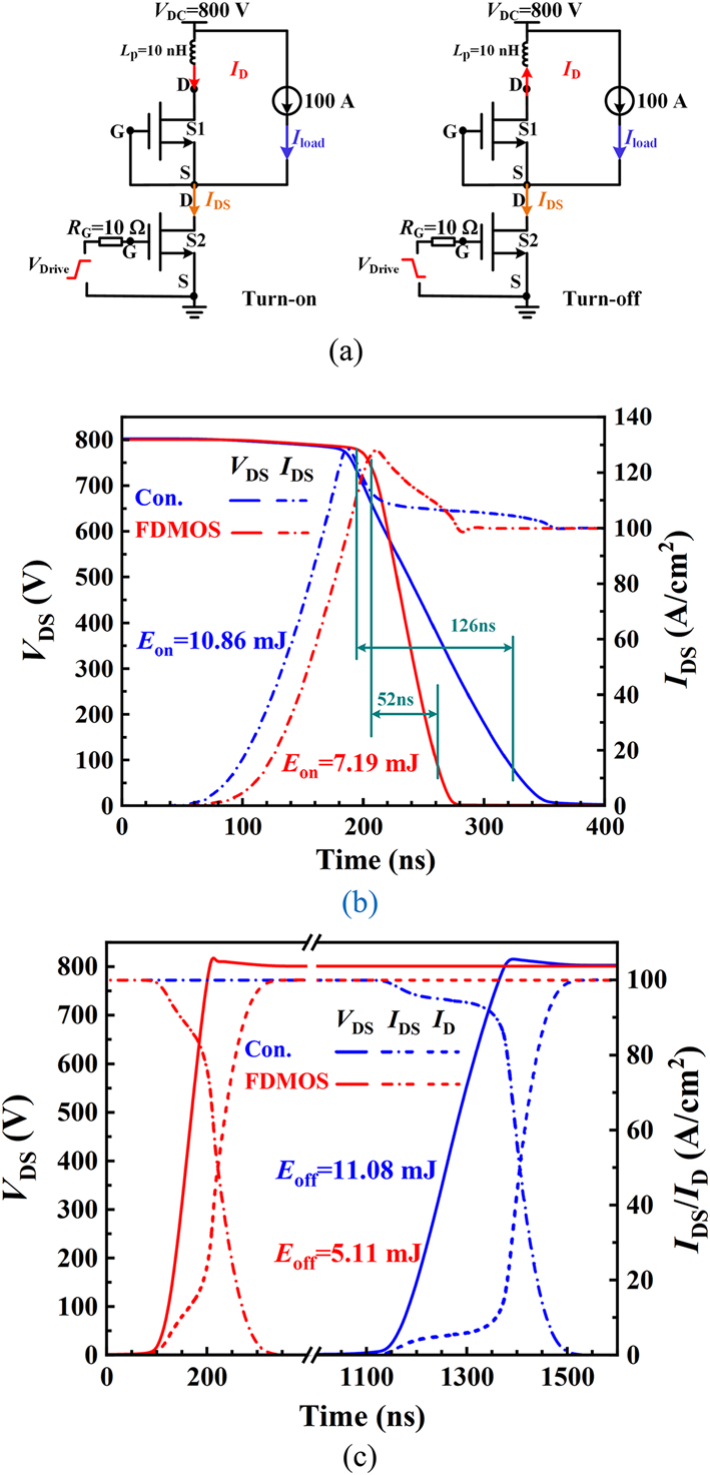
**a** Test circuit for turn-on/off process. P-i-N diode acts as FWD in the Con. MOS. Fin diode acts as FWD in the FDMOS. Simulation of the **b** turn-on and **c** turn-off transient for the FDMOS and the Con. MOS

Figure 8a shows the test circuit for switching characteristic. The Switch 1 (S1) and the Switch 2 (S2) are the same device, and they can be the proposed FDMOS or the Con. MOS. The gate and the source of the S1 is short-circuited as the FWD diode, and a parasitic inductor *L*_p_ = 10 nH is connected with S1 to simulate the overvoltage caused by reverse recovery of the FWD diode. Figure 8b, c shows power dissipation and turn-on/off curves. The *t*_r_ of the FDMOS and the Con. MOS are 52 ns and 126 ns, respectively. The current rise rate of the FDMOS and the Con. MOS is almost the same, and the voltage drop rate of the FDMOS is much higher than that of the Con. MOS due to the smaller *Q*_GD_. Therefore, the *E*_on_ of the FDMOS reduces by 33.8% in comparison with the Con. MOS. Owing to the low *Q*_GD_, as shown in Fig. 6, the *E*_off_ of the FDMOS reduces from 11.08 to 5.11 mJ, decreasing by 53.8% in comparison with that of the Con. MOS.

**Fig. 9:**
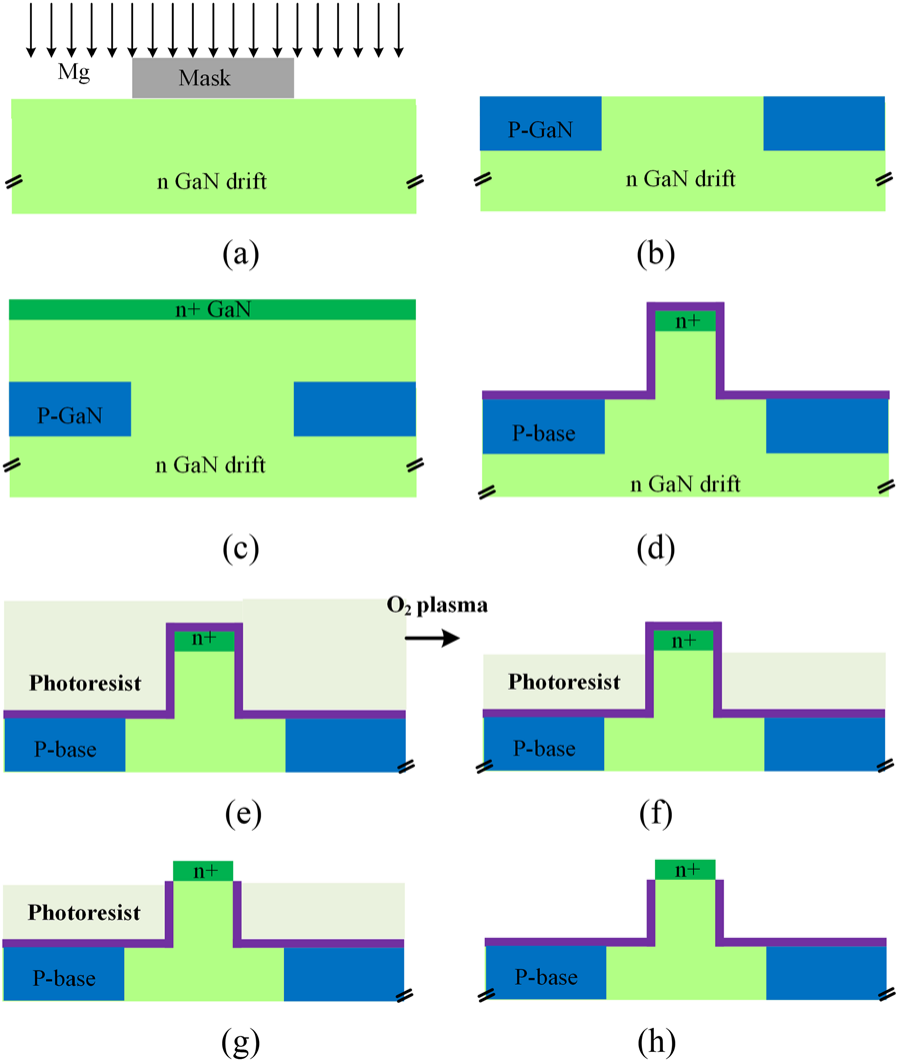
Key fabrication process flows for the FDMOS. **a**–**c** GaN epi layer growth [19]; **d** ICP etch to form the fin and deposit the Al_2_O_3_ dielectric; **e**–**h** Expose top n+ surface

The key process steps have been given out to show that the structure is doable, as shown in Fig. 9. The required GaN epi layer was proposed and fabricated in REF [19]. Firstly, the n-GaN drift region is grown by metalorganic chemical vapor deposition (MOCVD), and the P-GaN region is formed by implantation of Mg ion, as shown in Fig. 9a, b. Then, the top GaN layer is regrown by plasma-MBE. The p-GaN regions are used as the P-base region. Secondly, the fin is formed by Cl_2_/BCl_3_-based inductively coupled plasma (ICP) etching, and Al_2_O_3_ dielectric is formed by ALD, as shown in Fig. 9d. The oxide etch depth is controlled by a timed photoresist (PR) etch [20]. The mask is photoresist, treated with O_2_ plasma as shown in Fig. 9e, f. Then the Al_2_O_3_ dielectric is etched down by buffered oxide etch (BOE) to expose top n+ surface and the mask is removed, as shown in Fig. 9g, h. Complete FDMOS structure with implanted n+ source/drain, metal electrodes and anode metal is not drawn in Fig. 9.

## Conclusion

In this work, a normally off vertical GaN power MOSFET with an integrated fin-shaped diode is proposed. A unipolar fin-shaped diode realizes a low reverse conduction voltage and a low reverse recovery charge *Q*_rr_. Meanwhile, the design also results in a much smaller *Q*_GD_. Therefore, the FDMOS reduces the reverse turn-on voltage by 77.9% compared with that of the Con. MOS. The *Q*_GD_, *E*_on_ and *E*_off_ of the FDMOS are decreased by 56.8%, 33.8% and 53.8% respectively, compared with those of the Con. MOS. The integration of the fin-shaped diode eliminates the parasitic effect and reduces the total chip area, compared with the conventional method of using an external Schottky diode.

## Data Availability

All data generated or analyzed during this study are included in this article.
